# The hazard of large debris flows

**DOI:** 10.1126/sciadv.adz4625

**Published:** 2025-10-22

**Authors:** Erin L. Harvey, Tristram C. Hales, Alexander J. Horton, Oliver R. Francis, Fan Yang, Jie Liu, Xuanmei Fan

**Affiliations:** ^1^ School of Earth and Environmental Science, Cardiff University, Cardiff, UK.; ^2^ Department of Geography, Durham University, Durham, UK.; ^3^ Institute of Hazard, Risk and Resilience, Durham University, Durham, UK.; ^4^ Natural Resources Wales, Cardiff, UK.; ^5^ Klim, Berlin, Germany.; ^6^ State Key Laboratory for Geohazard Prevention and Geoenvironment Protection, Chengdu University of Technology, Chengdu, China.

## Abstract

Large (>10^6^ cubic meters), highly mobile debris flows represent one of the deadliest yet least understood types of landslides on Earth. These flows often originate when smaller events entrain water and sediment along their channel. The conditions controlling when and where these flows bulk are not well understood, making their hazard unpredictable. Here, we examine this hazard by combining a unique inventory of debris flows from the Wenchuan earthquake with numerical modeling to constrain their magnitude and frequency. We show that large debris flows occur more frequently than expected, on the basis of magnitude-frequency relationships for all debris flows, when high volumes of sediment are deposited in channels. These findings are consistent with other large sediment-generating events globally, such as Mount St. Helens and Mount Pinatubo where multiple large debris flows were triggered following volcanic eruptions that produced several cubic kilometers of sediment.

## Teaser

Large debris flows entrain sediment from extreme events like earthquakes and volcanic eruptions to form an escalating hazard.

## INTRODUCTION

Large, fast-flowing debris flows (including volcanic debris flows such as lahars) are a particularly catastrophic form of landslide, with single events leading to many thousands of fatalities (*1*) and destroying critical infrastructure (*2*, *3*). These highly saturated flows traverse channels tens of kilometers in length, inundate areas >10^5^ m^2^, and bulk to volumes that far exceed typical hillslope and channel debris flows [>10^6^ m^3^, defined as “large” flows by Major *et al.* (*4*)] (*4*, *5*). Prominent debris flows of this scale in the United States (Montecito, 2018) (*6*, *7*) and China (Wenchuan, 2019) (*8*) are expected to cost hundreds of millions USD for mitigation and recovery (*9*). The origin of these large flows remains poorly understood because these events are rare, often including only a single flow, and have a diverse range of triggering mechanisms and runout characteristics. The multihazard context in which flows of this magnitude occur, often as a part of a hazard cascade following volcanic eruptions, earthquakes, or tropical storms, makes it difficult to develop a systematic understanding of their potential hazard (*4*, *10*, *11*).

Debris flows are difficult hazards to predict in part because their size and velocity evolve as sediment and water are exchanged between the bed and the flow during transit (*5*). Where a debris flow traverses a dry, nonsaturated erodible bed, the entrainment of dry sediment increases internal flow friction, which slows the flow and reduces the runout length (*12*). Conversely, when debris flows traverse a saturated erodible bed, pore water pressures increase as the bed material is overridden and entrained in the flow (*13*). In this case, entrainment drives an increase in flow momentum by increasing the flow volume, through entrainment, and flow velocity, by reducing internal friction, to produce a highly mobile, sediment-laden debris flow (*12*). In physical experiments, this increase in flow momentum generates a runaway (positive) feedback effect such that the flow bulks until pore pressures decrease or sediment is exhausted (*12*, *14*). Recent physical and numerical models have simulated sediment-rich flows across an entrainable bed with varying water contents to replicate the increase in momentum as a function of bed pore water pressures (*15*–*17*). Horton *et al.* (*16*) varied the bed pore water pressure systematically to simulate debris flows triggered following the Wenchuan earthquake. The results indicate that specific bed conditions, particularly elevated pore water pressures, promote flow bulking. These conditions can lead to a bimodal distribution of debris flow volumes, with flow magnitudes increasing by an order of magnitude across a narrow interval of basal pore pressures. We refer to the specific set of conditions across which a debris flow exhibits pronounced bulking as the bulking threshold. We note that the conditions that drive the transition to rapid bulking are complex and not defined by a single, linear threshold. While these controlled physical and numerical experiments have demonstrated the presence of a runaway bulking feedback that could produce bimodal distributions of flow volumes, it has never been demonstrated in natural systems where complex flow and sediment conditions exist. Hence, it is possible that these large, mobile debris flows form a distinctive population of events that differ from smaller events where bulking is limited.

Analysis of the historical record of event magnitude and frequency is commonly used to calculate the potential hazard posed by debris flows (*1*, *18*). As with many other hazards, such as landslides and earthquakes, the shape of a debris flow magnitude-frequency curve is a power law for larger magnitudes (*19*, *20*). The magnitude-frequency relationship for debris flows is less well constrained and is often limited to smaller debris flows [e.g., <10^5^ m^3^ (*21*, *22*) or few events >10^6^ m^3^ on global scales (*23*)]. Therefore, we have been unable to determine whether large, highly mobile debris flows follow the same magnitude-frequency distribution as smaller debris or whether the bulking threshold identified in physical and numerical experiments causes large debris flows to fall along a different magnitude-frequency distribution. By conducting an event-scale hazard analysis using a database of debris flows triggered in the epicentral area of the 2008 Wenchuan earthquake, we seek to understand the hazard posed by large debris flows (*8*, *11*). We hypothesize that in natural systems, the positive momentum feedback that drives bulking in large, saturated debris flows is driven by the sediment and water availability along the beds of steep channels (*12*). The detailed annual mapping of debris flows following the Wenchuan earthquake provides a unique and comprehensive dataset from which to assess the hazard posed by large, saturated debris flows triggered under the same climatic, geomorphic, and topographic conditions. We generate a magnitude-frequency distribution for events that range from small (500 m^2^) to large (640,000 m^2^, assuming an average depth of at least 10 m to equate to >10^6^ m^3^). We test the role of channel sediment and bed pore water in controlling the magnitude-frequency distribution of debris flows using the numerical model Massflow (*15*). Last, we compare our findings with other large debris flow events (>10^6^ m^3^) to examine whether the dominant controls on large debris flows in Wenchuan are consistent globally.

## RESULTS

### Post–Wenchuan earthquake debris flows

The 2008 *M*_w_ (moment magnitude) 7.9 Wenchuan earthquake generated ~3 km^3^ of landslide sediment in one of the most voluminous earthquake-triggered landslide events in recent history (*24*). A decade after the earthquake, up to 90% of this sediment remained on hillslopes and in low-order drainage basins. Postearthquake landslides and debris flows have formed a major sediment cascade by remobilizing up to 10% of this coseismic landslide material downslope into tributary channels and the Min River (*25*).

**Fig. 1.**
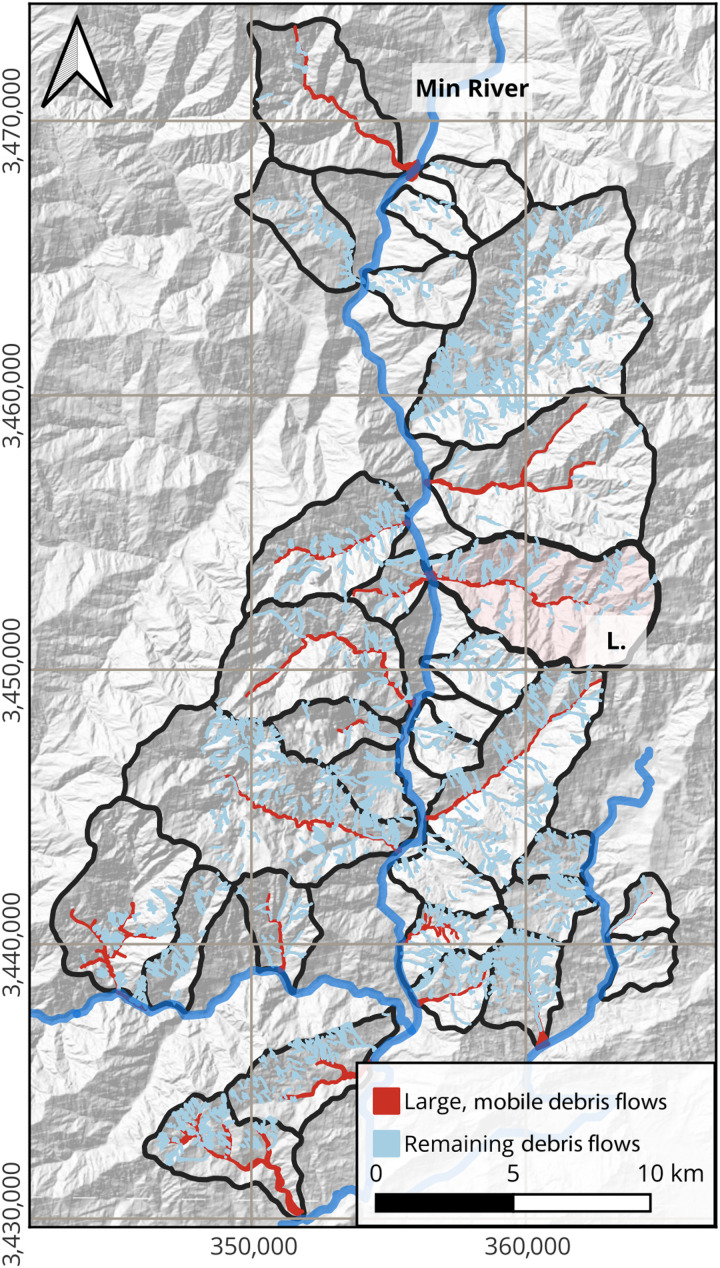
Wenchuan, China, with the 2058 debris flows and 31 catchments used in this study outlined. Catchments were selected on the basis of their comprehensive and detailed debris flow records. Polygons represent the debris flow inventory for 2008 to 2019, with large, mobile debris flows in red and all other debris flows in blue. The Luoquanwan catchment, shaded in pink and labeled L., is used for the numerical modeling component of our study. The Min River is flowing from north to south.

**Fig. 2.**
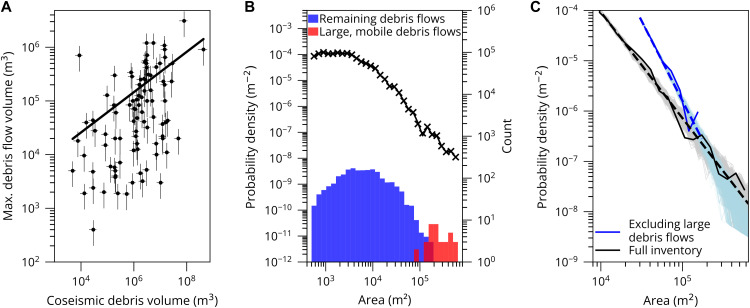
Analysis of the post–Wenchuan earthquake debris flow inventory. (**A**) Volume of coseismic landslide debris deposited in the catchment plotted against the largest postearthquake debris flow recorded in the catchment. Volume of coseismic landslide material within 92 catchments using the landslide inventory by Li *et al.* (*24*). The volume was calculated by averaging several established area-volume scaling relationships (*24*, *54*–*56*), and error bars show the standard deviation of those estimates. Volumes of stored coseismic debris are plotted against the maximum debris flow volume for each catchment taken from Fan *et al.* (*26*). We applied a conservative error of 50% to these estimates. (**B**) Magnitude-frequency distribution of all debris flows in our inventory as a probability density function (*N* = 2058 and *x*_min_ = 500 m^2^ to show the complete dataset). The counts of large and all remaining debris flows as histograms are plotted on the secondary *y* axis in red and blue, respectively. Large debris flows (red) are overlaid onto the histogram of all remaining debris flows; darker shading therefore represents overlap. The histogram is binned on a logarithmic scale. (**C**) Magnitude-frequency distributions for all debris flows in our inventory (*N* = 2058 and *x*_min_ = 8500 m^2^ to fit with the power law; Materials and Methods) and excluding large, mobile debris flows (*N* = 2021). Dashed lines show lines of best fit (all debris flows, exponent of 2.1 with standard error ± 0.04; remaining debris flows, exponent of 3.1 with standard error ± 0.17) for both distributions. The pale blue and gray lines represent 1000 distributions created by bootstrap-sampling 2058 points from the best-fit distributions.

Debris flow activity is well documented after the Wenchuan earthquake (*26*). The largest debris flows generated deposits with areas up to 640,000 m^2^ and traversed several stream orders (Fig. 1) (*8*, *26*). Extensive debris flow mapping identified 2044 debris flows (>500 m^2^ area) between 2008 and 2018 over a study area of 416 km^2^ (*26*). We supplement this dataset with 14 debris flows triggered in 2019 (*8*) to create a dataset of 2058 flows. Included within this dataset are multiple large debris flows that were triggered in catchments along the Min River in 2008, 2010, 2013, and 2019 (*8*, *11*, *27*). These debris flows (locally referred to as catastrophic debris flows) were characterized as extremely mobile and able to transit both low- and high-order streams and ceased only when reaching a much higher-order river, such as the Min River (Materials and Methods and Fig. 1) (*8*, *28*). We found that 37 debris flows fit these characteristics and refer to these as large, highly mobile debris flows, because their characteristics suggest that they experienced pronounced bulking. These flows occurred between 2008 and 2019 across 18 catchments, with 11 catchments having multiple large flows. There was no clear relationship between the size of large, mobile debris flows and the maximum slope or catchment area, suggesting that the distribution of these flows is not simply topographically constrained (figs. S1 and S2). However, there is a relationship between the amount of coseismic landslide debris within catchments and the total volume of the largest debris flow in the catchment (Fig. 2A), consistent with previous observations of a correlation between sediment stored within debris flow channels and their magnitude (*27*).

### Magnitude-frequency relationships for large debris flows

There is a distinctive increase in the frequency of events greater than ~100,000 m^2^ relative to smaller events in the magnitude-frequency distribution of Wenchuan debris flows (Fig. 2B). This change in frequency of the largest flows correlates with the 37 large, mobile debris flows and infers that these debris flows occur at a higher frequency than would be anticipated by extrapolating the magnitude and frequency of smaller debris flows (Fig. 2B). To determine whether the apparent increase in large events could be due to randomness in the extreme value distribution, we used the Python powerlaw package to fit a power law on the basis of maximum-likelihood fitting to the magnitude-frequency distribution for all debris flows mapped in the inventory (*N* = 2058) (*29*, *30*). We used this power-law distribution to bootstrap sample random distributions of 2058 flows. We replicated this bootstrap sampling 1000 times to determine the range of possible magnitude-frequency distributions that could be derived randomly from the best-fit power law (Materials and Methods and Fig. 2C). We found that the change in frequency of events larger than 100,000 m^2^ was at the extremes of the random sample distribution. As a secondary test, we repeated the same bootstrapping method for all remaining debris flows. Extrapolation of the data for remaining debris flows only generated a power-law relationship with a steeper gradient than the complete dataset (Fig. 2C). We acknowledge the limitations of using power laws for relatively small sample sizes and the challenges of overinterpretation (*31*). To minimize this, we noted that there are fewer observations of debris flows in total with areas of 10^5^ m^2^, the point at which we argue for a process transition. First, we revisited the imagery used to define the original dataset but were unable to find evidence that we had misidentified flows. In addition, we tested whether this was a statistical anomaly by randomly resampling our data and varying bin widths (fig. S3 and Materials and Methods). The change in frequency at ~10^5^ m^2^ persists in different choices of bin width apart from when unrealistically small (<10) numbers of bins were chosen. On the basis of these tests, we demonstrate that it is unlikely that the change in frequency of debris flows could be attributed to systematic errors in the original dataset or our application of statistical methods. Rather, we interpret the data as showing that large debris flows do not fall on the same continuum as smaller debris flows. Thus, large debris flows occur more frequently than expected in Wenchuan, causing a marked underestimation of their hazard.

**Fig. 3.**
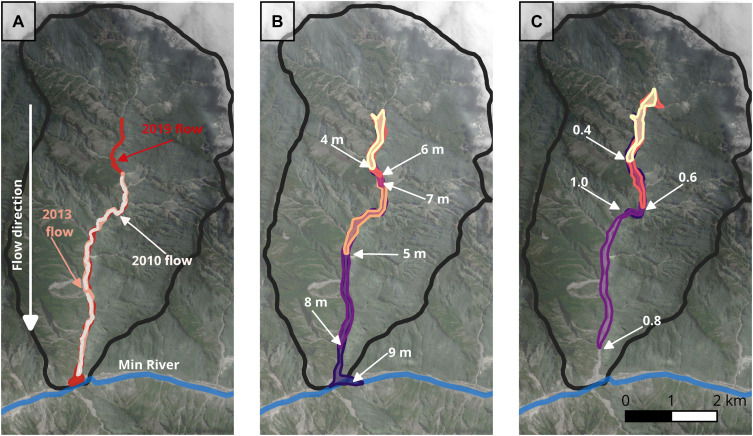
Large debris flow model simulations for the Luoquanwan catchment. (**A**) Extent of three large debris flows that occurred within the Luoquanwan catchment. Events are labeled with the year in which they occurred. The source location for the 2019 event is used to define the location for the source volume in Massflow. (**B** and **C**) Simulated debris flows using Massflow within the Luoquanwan catchment. All flows were triggered using a source volume of 172,800 m^3^. In (B), all parameters remained the same (degree of bed saturation of 0.8), and only the depth of erodible sediment changed. Arrows are used to show the runout of debris flows on the basis of the input depth of erodible sediment. In this example, the simulation required an erodible depth of 8 or 9 m to reach the end of the catchment. In (C), we vary the degree of bed saturation; in this case, only the simulation with a degree of saturation of 0.8 traveled near to the end of the catchment. Here, we use a depth of 8 m for all simulations.

We hypothesize that the deviation of debris flow frequency with size is because of a set of conditions that drive substantial bulking along the debris flow path. We test this using a two-dimensional (2D) debris flow runout model, Massflow, calibrated to a large debris flow event that occurred in 2019 in the Luoquanwan catchment (Figs. 1 and 3A) (*8*, *16*, *32*). Massflow simulates sediment and water mixtures flowing across an erodible bed including the entrainment and bulking of the debris flow mass. We simulate 168 debris flows by systematically varying the depth of sediment along the flow path, the relative degree of bed saturation, and the volume of sediment initially mobilized to identify critical controls on the development of large debris flows (Fig. 3A). We varied the erodible sediment depth at 1-m intervals between 4 and 10 m (*8*). Source volumes included four possible coseismic landslide volumes that fall within the distribution of landslide volumes observed in Luoquanwan (24,300, 97,200, 172,800, and 270,000 m^3^) (*26*). Last, the degree of channel bed saturation varied from 0 to 1 at 0.1 increments (*16*).

**Fig. 4.**
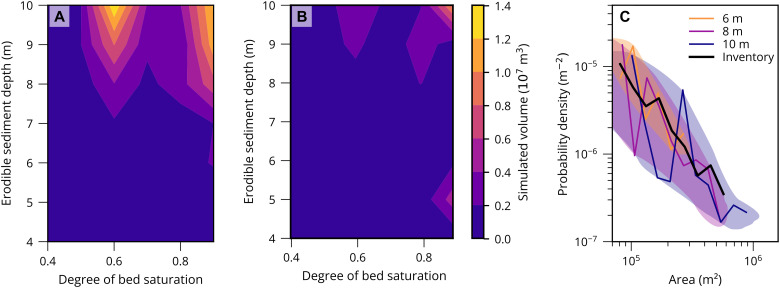
Controls on simulated debris flow volume for Massflow runs. (**A**) Relationship between the degree of bed saturation and the erodible sediment depth relative to the simulated debris flow volume. All simulated runs have a source volume of 270,000 m^3^. (**B**) Relationship between the degree of bed saturation and the erodible sediment depth relative to the simulated debris flow volume. All simulated runs have a source volume of 172,800 m^3^. The color bar shown applies to (A) and (B). The two smallest source volumes did not produce any debris flows that reached the catchment outlet, and so, they are not shown. (**C**) The magnitude-frequency distributions for simulated debris flows in the Luoquanwan catchment when varying the depth of entrainable sediment (6, 8, and 10 m) and assuming the source volume and the degree of saturation follow a uniform distribution (Materials and Methods). Magnitude-frequency distributions were generated as probability density functions using the powerlaw Python package and an *x*_min_ of 70,000 m^2^. Envelopes are used to emphasize the general trends observed from each curve. The inventory dataset from Wenchuan is represented by a thick black line.

To produce a debris flow that traversed the Luoquanwan catchment, a narrow set of conditions was required (Fig. 3). Most flows with a simulated volume of at least ~2.5 × 10^6^ m^3^ reached the catchment outlet (Fig. 3). In general, to simulate a debris flow that reached the catchment outlet, large source volumes, sufficient in-channel sediment storage, and a high degree of bed saturation were necessary (Fig. 4). The degree of bed saturation was critical to developing a large and mobile debris flow, with large, mobile debris flows only generated when the degree of bed saturation equated to 0.8 and 0.9. The bed saturation needed for large debris flows in Luoquanwan is slightly higher than observed for simulations of the Hongchun debris flow, a large, mobile debris flow that traversed only first- and second-order channels (*16*). We appreciate that the conditions required to generate a large debris flow are complex and nonlinear; for example, for a fully saturated bed (1.0), the simulated debris flows are large but do not reach the catchment outlet. Similarly, the large debris flow generated with a bed saturation of 0.6 did not reach the catchment outlet (Fig. 4A). Once the bed is sufficiently saturated, the size (length and volume) of the resulting debris flow depends on the depth of available bed sediment, denoted by the larger simulated volumes for 10-m sediment (Fig. 4). Once this threshold is exceeded, the length and volume of the flow are limited by whether the sediment entrained by the flow is of sufficient volume to maintain downslope movement. Hence, where the sediment thickness in the channel was relatively thin (<5 m in the model), the debris flows lost momentum before reaching the outlet of the Luoquanwan catchment, as shown by the consistently low simulated volumes for 4-m sediment (Fig. 4). These simulations demonstrate that for a debris flow to reach the catchment outlet in Luoquan, it must bulk via the entrainment of bed material. This entrainment is limited by bed pore pressures, the source volume, and the depth of available in-channel sediment (*16*, *33*). The positive momentum feedback effect is therefore a probable explanation for the high magnitude of the debris flows observed in Wenchuan (*12*).

To understand how channel sediment affects the hazard associated with large, mobile debris flows, we created 10 synthetic magnitude-frequency distributions by sampling randomly from our model runs. To generate each simulated magnitude-frequency distribution, we ran a Monte Carlo simulation to obtain 2000 simulated debris flow volumes controlling for in-channel sediment depth but varying the sample distribution for other variables (source volume and bed saturation). For each simulation, parameter values were chosen at random, assuming a uniform distribution, or were sampled from an empirical distribution (table S1). We convert the total volume to total area to minimize the effects of pooling in model runs where debris flows were not triggered. We then use the area to compare the simulated magnitude-frequency distributions to the observed Wenchuan magnitude-frequency distribution. Our model simulations most closely reproduce the magnitude-frequency distribution seen in Fig. 4C at an in-channel sediment depth of 8 m, and where the degree of bed saturation and source volume are selected from a uniform distribution, such that the sediment thickness limits the potential for large debris flows.

Hillslope sediment stores play an important role in generating sufficient channel sediment, particularly in steep, monsoonal landscapes where it is possible to generate sufficient bed saturation values, such as Wenchuan. For example, in the Wenchuan region, extensive earthquake-triggered landslide deposits are being reworked by channel and hillslope processes (Fig. 2A) (*25*). Field observations have also shown a shift in the locus of triggering of large debris flows from hillslopes to channels (*8*, *27*). Similarly, a single large landslide in Wenchuan alone would not generate a large debris flow; the flow must also bulk during transit and therefore is reliant on sufficient channel sediment (fig. S4).

## DISCUSSION

We demonstrate that large debris flows triggered following the Wenchuan earthquake only reach the catchment outlet through the self-sustaining positive momentum feedback effect. This feedback effect requires sufficient pore pressures, a large source volume, an abundance of entrainable in-channel sediment, and substantial in-channel topography, conditions that rarely co-occur in nature (Fig. 3). These conditions are more commonly achieved in Wenchuan because of the abundant coseismic material that both acts as a source material (*34*) and has been transported to debris flow channels by postearthquake processes (*25*). The annual monsoon also generates sufficient precipitation that can saturate channel material (*35*) and trigger debris flows. Field observations also found that the deposits of these flows were well mixed, suggesting that flows were highly saturated (*28*). The confluence of large volumes of sediment within steep mountainous topography supports the development of large debris flows frequently enough to examine their statistical properties.

While many mountain ranges are sediment poor (*36*), single large sediment-generating events can substantially increase the consequent hazard posed by large debris flows in a short space of time. Mountainous bedrock landscapes commonly experience debris flows yet rarely experience large debris flows even when there are substantial relief and frequent hydrological triggering conditions such as monsoon rainfall. Our empirical data from Wenchuan and modeling highlight that where channel sediment volumes are small, the lack of entrainable sediment along the flow path limits their size. Recent data from the 2021 Melamchi debris flood have shown the entrainment of in-channel sediment in the headwaters of the flow (*37*, *38*). Our research moves beyond the widely accepted notion that the sediment depth can control the timing, location, and volume of debris flows (*39*–*41*) to show that in areas where there are an abundance of sediment and the culmination of a narrow range of conditions, debris flows have the potential to bulk catastrophically. The erodible sediment depth should therefore be considered as a first-order constraint on the magnitude and frequency of large debris flows.

The occurrence of multiple large debris flows in close spatial and temporal proximity is relatively rare, with only three other events of a similar scale (>10 large debris flows) documented historically: Mount Pinatubo, Vargas, and Mount St. Helens (*4*, *42**,* *43*). The volume and rheological characteristics of the Mount St. Helens lahars have been recorded in detail (*4*, *8*), but the large number of debris flows (lahars) in the Pinatubo example is recorded qualitatively or as overall sedimentation rather than by count (*42*). In Wenchuan, Mount Pinatubo, and Mount St. Helens, the large number of debris flows occurred within a decade of an event that produced a large amount of sediment (>3 km^3^), either by earthquake-triggered landsliding or volcanic ashfall (*4*, *25*, *42*, *44*). Qualitative observations of the several large debris flows triggered in the decades following the eruption of Mount St. Helens have described flows as turbulent, sediment-rich, and coarser than hyperconcentrated flows but with less structure than a debris flow (*4**,* *45*). These flows were also found to entrain up to 90% of their sediment during transit. These observations indicate that the hazard posed by large debris flow events may be elevated for several decades as part of the hazard cascade of these larger events (*4*). The hazard posed by large, mobile debris flows is likely to cease as sediment stores stabilize, such as through the removal of sediment or revegetation of deposits (*46*); however, further research is necessary to gauge the potential for large debris flows in these systems beyond several decades.

Single debris flow events can also provide support for the relationship between large debris flow generation and in-channel sediment. Notably, a smaller debris flow that would not fit our definition of a large debris flow (230,000 m^3^), triggered by a glacial lake outburst flood in Norway, was shown to increase in volume by a factor of 10 through entrainment (*47*). This flow had very similar characteristics to the flows we have described, including large boulders, turbulent flow, and an unusually long runout. This study predates the positive momentum feedback effect; however, the authors inferred that the unusually large bulking and runout were likely related to the volume of water released by the glacial lake outburst flood as well as the abundance of loose sediment along the channel. This study suggests that the shift in magnitude-frequency that we observe may uniquely describe the effect of the positive momentum feedback effect in Wenchuan, and to fully capture the characteristics of and hazard posed by rapidly entraining debris flows, more regional scale assessments are needed.

We demonstrate that well-documented past events provide unique insight into mechanisms controlling debris flow runout and magnitude. While large debris flows are rare in nature, there is a set of consistent rheological characteristics, such as being highly mobile, saturated, and sediment-rich, that can govern their potential hazard. In Wenchuan, we characterized these flows as channelized flows that traverse several stream orders, typically with source locations in headwater channels, before depositing when joining a much larger river. These characteristics can be adapted for locations and inventories beyond Wenchuan to produce quantitative regional assessments that explore the magnitude and frequency of large, mobile flows over several decades (*48*). Where sediment is not limiting, such as in Wenchuan, not only do large debris flows form, but they occur at a frequency that is at the upper end, or higher, than would be expected from the extrapolation of magnitude-frequency relationships from previous events. As such, large debris flows represent a prominent source of epistemic uncertainty when considering the secondary cascading hazards after large stochastic earthquakes or volcanic eruptions. Our work demonstrates that simple analyses of sediment volumes and their spatial distributions after large earthquakes may provide a coarse tool for estimating the potential of future large debris flows. The positive relationship observed between coseismic landslide volumes and maximum debris flow volumes provides an initial benchmark for this in Wenchuan (Fig. 2A). Beyond this, constraints on sediment depth and pore pressures in flows would provide further guidance on the potential for large debris flows in catchments.

Where sufficient sediment and water are available in steep catchments, channels have the risk of developing large, mobile debris flows. The rapid entrainment by these highly mobile, sediment-rich debris flows far exceeds most hillslope and channel debris flows and can increase the magnitude of the flow and, thus, potential hazard, with runouts extending tens of kilometers in length. We demonstrate that by considering large debris flows with the same magnitude-frequency power law as typical debris flows in Wenchuan, we are underestimating the hazard posed by these large, destructive flows. Hazard models should therefore reconsider how the relatively high frequencies of these large flows will affect risk to communities. From other historical events, we infer that large debris flows are not necessarily related to the initial sediment-generating event (e.g., wildfires, earthquakes, or volcanic eruptions) or trigger (e.g., rainfall and outburst flood) but instead to the amount of sediment available in the landscape. Future events that expose sediment either via a stochastic source (e.g., an earthquake) or through climate change–driven glacial retreat may drive an increase in future large debris flow events.

## MATERIALS AND METHODS

### Wenchuan debris flow inventory

We analyzed the multitemporal inventory developed by Fan *et al.* (*26*), which contained 2044 active debris flow events >500 m^2^ between 2008 and 2018. We updated the inventory to include several additional debris flows triggered in August 2019 (*N* = 2058) (*8*). We used metrics such as stream order, deposit location, and field observations to identify large debris flows from the multitemporal inventory based on our criteria, in which large debris flows were highly mobile with large runout lengths and only ceased when entering the Min River. Most large debris flows traversed at least three stream orders before depositing into a fourth-order or higher channel, such as the Min River (*28*). Stream orders for the study region were obtained using the Strahler method and a 200-pixel flow accumulation limit for a 30-m digital elevation model. Debris flows in catchments that were extremely steep and therefore were not clearly channelized, were too small, or had no debris flows were removed, leaving 31 catchments for our analysis (Fig. 1).

### Debris flow inventory magnitude-frequency distributions

We derived a magnitude-frequency distribution using total flow plan-view area for the individually mapped debris flows and fitted a power-law function using the Python powerlaw package (*29*). We used area to minimize errors associated with the debris flow depth, which can often be highly variable and difficult to accurately measure, being carried forward in our empirical stage of analysis (*25*). Errors of amalgamation, which can be found in manually mapped landslide inventories, were minimized by manually splitting merged flows where imagery was available (*49*). If imagery was not freely available to validate the polygons, we considered the flows as a single flow. Most merged debris flows were not considered large debris flows, and therefore, it is unlikely that our findings will be affected.

The powerlaw package uses the mathematical method developed by Clauset *et al.* (*30*) to determine whether a power law can be fitted to a dataset. Where possible, we selected the power law with the lowest *D* value, which represents the minimal Kolmogorov-Smirnov distance between the data and the fitted line, and the lowest σ value, which is the standard error and is <0.05 for the entire inventory and the subset of the inventory, which excluded large debris flows. The small number of large debris flows in comparison to the other inventories meant that this threshold was not attainable.

The power law for the entire inventory has an exponent of 2.1, which describes the slope of the power law, and an *x*_min_ of 8500 m^2^, which describes the minimum area to which the power law can be fit (Fig. 2C). The tail of the distribution contains 36% of debris flows (*x* > *x*_min_). The power law for the dataset, excluding large debris flows, was steeper with an *x*_min_ of 30,000 m^2^ and an exponent of 3.1 (Fig. 2A). A test determining whether a lognormal or power-law distribution was best fit was insignificant (*P* > 0.05).

To assess the effect of including large debris flows in the full magnitude-frequency distribution, we used a Monte Carlo analysis. In this analysis, we used the equations for the power law derived using all postearthquake debris flows and postearthquake debris flows excluding large, mobile debris flows to simulate 1000 magnitude-frequency distributions. Each magnitude-frequency distribution consisted of 2058 values, consistent with the number of debris flows in the Wenchuan inventory. We overlaid these distributions onto the real distribution from the multitemporal inventory to determine whether the change in slope observed at 100,000 m^2^ was an artifact of the 2058 debris flows within the sample (Fig. 2C).

The use of power-law distributions to represent natural processes remains challenging and can lead to the overinterpretation of datasets (*31*). We chose to generate additional probability density functions to determine whether the change in frequency observed for large debris flows relates to the chosen bin width or data used. We therefore varied the proportion of data included (from 50 to 100% at 10% increments) and the number of logarithmic bins that the data are divided into (from 5 to 200). We generated probability density functions on the basis of different bin widths (fig. S3A). We find that the shift in frequency observed in Fig. 2B is prevalent when the number of bins is set to at least 10. This value fits with the recommended number of bins, which is between 10 and 32 depending on whether we use Bayesian blocks (*50*), Doane’s formula, or the bin width in the powerlaw package (*29*). We also find that the shift in frequency for large debris flows is visible when using only 50% of the dataset, selected by random (fig. S3B).

### Simulating debris flow volume in Massflow

We used the 2D debris flow model Massflow to simulate a large debris flow event in the Luoquanwan catchment using the source location of the 2019 large debris flow for reference (Fig. 3A). The debris flow covered an area >420,000 m^2^ and was the focus of a detailed field study in (*28*). The model has been previously used to model postearthquake debris flows in Wenchuan and to demonstrate that a critical saturation ratio for bed material was required to produce extremely large debris flows in the Hongchun catchment (*16*).

Massflow is a 2D depth-integrated debris flow model (*15*). Massflow was developed to simulate large mountain hazards, such as debris flows and dam break floods, over the natural terrain at a high resolution and with relatively low computational intensity. An important component of Massflow, which makes it well suited to the analyses conducted here, is the ability of the simulated flow to entrain sediment from a static bed, which also has a set of input characteristics. The derivation of the governing equations has been published in full detail in (*15*, *32*). The shallow water equations are solved using a second-order MacCormack-TVD finite difference method. The model is run in a rectangular global Cartesian frame, which has been rotated so that it is parallel to the slope angle.

We use a modified version of Massflow, which considers the entrainment rate of the flow (*16*). The entrainment rate must satisfy the following boundary condition, where the density of the flowing layer, ρ_1_, is constant and topography is neglected (Eq. 1)

$$E=-\frac{\partial z_{1\text{bot}}}{\partial t}=\frac{\tau_{1b}-\tau_{2s}}{\rho_{1}\sqrt{u_{1}^{2}+}v_{1}^{2}}$$ (1)

where $u_{1}$ and $v_{1}$ represent depth-averaged velocities in the *x* and *y* directions for the flowing mass, respectively; $\rho_{1}$ represents the density of the flow; $z_{1\text{bot}}$ is the basal boundary between the flow and the static bed; $\tau_{1b}$ is the basal traction of the flow; and $\tau_{2s}$ is the resistive shear stress from the erodible layer. The model uses both Coulomb and Voellmy friction laws when quantifying the basal traction of the flow (Eq. 2). By combining both friction laws, the model can simulate the flowing and stopping mechanisms associated with debris flows. For example, the Coulomb friction law does not consider flow velocity, which is thought to be an important control on entrainment (*5*), and the Voellmy model has a smaller friction angle than the angle of repose. Previous studies have used an assigned critical velocity to encourage a debris flow modeled using a Voellmy simulation to stop (*15*). Therefore, the combined model can capture the increase in traction with velocity without decreasing the friction angle

$$\tau_{1b}=\text{max}\left[\rho_{1}g_{z}h_{1}\text{tan}(\varphi_{\text{voellmy}})+\frac{\rho_{1}\left(u^{2}+v^{2}\right)}{C_{z}^{2}},\rho_{1}\left(1-s\right)g_{z}h\text{tan}\left(\delta \right)\right]$$ (2)

Here, $s=\frac{\rho_{w}}{\rho }$ , $\varphi_{\text{voellmy}}$ is the internal friction angle of the flowing mass, $g_{z}$ is the gravity acting normal to the inclined slope, $C_{z}$ is the Chezy coefficient (which is a function of flow Reynolds number and the relative roughness of the channel), δ is the basal friction angle, and $h_{1}$ is the flow depth. $\varphi_{\text{voellmy}}$ , $C_{z}$ , δ , and ρ are all parameters input into Massflow by the user.

A Coulomb failure criterion is used to describe the total resistance stress from the bed, where

$$\tau_{2s}=c+\rho_{1}g_{z}h_{1}\left(1-\lambda \right)\text{tan}\left(\phi_{2}\right)$$ (3)

*c* is the cohesion of the bed material, $\phi_{2}$ is the internal friction angle of the bed material, and λ is the pore water ratio (or the degree of bed saturation).

We ran a sensitivity analysis on all parameters in Eqs. 2 and 3 to determine which parameters we should vary to better understand debris flow entrainment in the Luoquanwan catchment. The primary control on the output volume and runout was the pore water ratio (*16*). We also varied the source volume, which was of secondary importance in the Hongchun catchment (*16*), and the sediment depth. All other variable values had no systematic effect on debris flow runout for the varying source volumes and pore water ratios and were therefore kept consistent with the values used previously (table S2) (*16*). The only variable that changed between Hongchun (*16*) and Luoquanwan (this study) was the basal friction angle. In this study, a basal friction angle of 20° was necessary to produce a large debris flow, which reached the catchment outlet, whereas in Hongchun, a value of 28° was sufficient.

We ran Massflow, varying the three parameters (source volume, sediment depth, and degree of bed saturation/pore water ratio) in a systematic approach. We used four input volumes: 24,300, 97,200, 172,800, and 270,000 m^3^. These volumes were chosen on the basis of the volumes of coseismic landslides in Luoquanwan and represent approximately the 20th, 60th, 80th, and 90th percentiles of the distribution based on an arbitrary depth of 3 m. We varied the depth of channel bed sediment by varying the amount of material that could be eroded during each time step. We used static values to represent the depth of sediment that could be entrained from 4 to 10 m at 1-m intervals, which are consistent with field observations of large debris flows in Wenchuan and elsewhere (*4*, *8*). On the basis of the sensitivity analysis above, we varied the pore water ratio, which gives an indication of how saturated the bed material is, at 0.1 increments from 0.4 to 1.0. From initial testing, we found that the change in debris flow volume and area between pore water ratios of 0 and 0.5 was negligible and therefore used the runs for a pore water ratio of 0.4 to represent these values. In total, we ran Massflow 168 times, varying each parameter in turn, to obtain 168 debris flow volumes.

Debris flow volume was the primary output from Massflow. While we could measure the area from the 2D flow simulation, we noticed that in some simulations with low pore water ratios, where small debris flows were triggered, the material in Massflow pooled and, in some instances, flowed upstream for several meters. When the degree of bed saturation was set to 1.0, we also found that Massflow simulated flows with large volumes that did not travel to the end of the catchment (see Fig. 3C). To minimize the overestimated areas for smaller simulations, we used the output volumes and areas to produce a volume-area scaling relationship. We used the scaling relationship to infer a more conservative estimate for debris flow area from the simulated volumes (fig. S5). These areas can then be compared with the field inventory, which considers debris flow magnitude using area. Previous debris flow volume-area scaling relationships did not fit the dataset well, which may in part relate to the fact that all the debris flows modeled here had an area >37,000 m^2^ and a volume >23,000 m^3^, whereas in Griswold and Iverson’s work (*51*), a wider range of volumes (from 10 to 10^7^ m^3^) was considered. The power-law relationship used to describe the relationship between the area and the volume was $A=101V^{0.55}$.

### Simulated debris flow magnitude-frequency distributions: Monte Carlo analysis

For our simulated debris flow magnitude-frequency distributions, we used either a uniform distribution or empirical distribution to select the value for each of the three parameters (table S1). The empirical distributions were based on field or remotely sensed data from within the Luoquanwan catchment and used to provide a more realistic set of values for the input parameters. The distributions chosen to describe pore water ratios and source volume are explained in more detail below. Because of the lack of field data, we did not vary the sediment depth on the basis of an extreme value parameter and instead used single values (e.g., 6, 8, and 10 m) as opposed to the full distribution. When using a random uniform distribution, each discrete input parameter value had an equal probability of being selected. All parameters were discrete, meaning that there were 11 pore water ratio values to choose from, 4 source volumes, and 7 depths. There were 308 possible combinations of parameter values, of which 168 values corresponded to unique debris flow volumes, given that runs for pore water ratios between 0 and 0.5 were assumed the same and therefore all inferred on the basis of running the model with a pore water ratio of 0.4. On the basis of either an empirical or uniform distribution, we selected three parameter values and the corresponding simulated debris flow volume. We repeat this process 2000 times as part of a Monte Carlo simulation to produce a magnitude-frequency distribution. The 10 Monte Carlo simulations were compared to assess whether uniform distributions or empirical distributions produced a magnitude-frequency relationship consistent with the distribution observed in the multitemporal debris flow inventory (Fig. 4C and fig. S4).

Precipitation was used to infer pore water ratios on the basis of the assumption that intense precipitation values correspond to a higher degree of bed saturation. Precipitation is a well-established control on debris flow initiation, and intensity-duration thresholds are typically used to predict events in which a debris flow may occur. Daily precipitation values for the Luoquanwan catchment were downloaded from the IMERG (Integrated Multi-satellitE) Global Precipitation Model (*52*). To obtain the daily rainfall in Luoquanwan, we averaged two 10-km by 10-km cells that overlapped the catchment. We grouped the daily rainfall into rainfall events. Here, a rainfall event is defined as a period of consecutive days in which rainfall occurs. The beginning and end of each event are determined by a day with no rainfall, defined using daily (24-hour) rainfall records downloaded from IMERG. For each rainfall event, we calculated the maximum daily rainfall. We removed all events with a maximum daily rainfall below the average 24-hour intensity-duration threshold for debris flow initiation in Wenchuan (26.1 mm) (*53*). This ensured that we only considered events above the rainfall threshold to trigger a debris flow. We used the Python package Fitter to determine whether a lognormal distribution best represented the maximum daily rainfall events in Luoquanwan. We then normalized the rainfall values by the largest maximum daily rainfall value so that the largest precipitation event was represented by a value of 1 and the values closest to the triggering threshold had a value just above 0. These normalized values were assumed to directly correspond to the pore water ratio value input into Massflow, whereby the highest rainfall events corresponded to the highest pore water ratio values. While this is an oversimplification, our model always simulated a debris flow, and therefore, we wanted to ensure that only rainfall values where this was possible were considered. There were 11 potential values for pore water ratio, ranging from 0 to 1 at intervals of 0.1.

We use an inventory of coseismic landslides within the Luoquanwan catchment to infer source volumes as these are likely to be one of the main sources from which debris flows initiate in Wenchuan (Fig. 2A) (*25*, *26*). Assuming a depth of 3 m, we convert areas to volumes and fit an inverse gamma distribution using the Python Fitter package. We then sample randomly from this distribution and select the source volume closest to the random value. There were four possible source volumes to choose from based on our model runs; we use four because of time constraints when running the model. We represented the maximum sediment depth that could be entrained using both randomly distributed values between 4 and 10 m, in increments of 1 m, and specific uniform depths (6, 8, and 10 m) to infer how the maximum sediment depth that could be entrained influenced the distribution of debris flow volumes.
