# Supplementary material for: The hazard of large debris flows
Source: Sci Adv. 2025 Oct 22;11(43):eadz4625. doi: 10.1126/sciadv.adz4625 (PMC12542927; doi:10.1126/sciadv.adz4625)
Supplement: Supplementary file 1 — Figs. S1 to S5 Tables S1 and S2 [file sciadv.adz4625_sm.pdf]

Science Advances
AAAS

Supplementary Materials for

The hazard of large debris flows

Erin L. Harvey *et al.*

Corresponding author: Erin L. Harvey, erin.l.harvey@durham.ac.uk; Xuanmei Fan, fxm_cdut@qq.com

Sci. Adv. **11**, eadz4625 (2025)
DOI: 10.1126/sciadv.adz4625

This PDF file includes:

Figs. S1 to S5
Tables S1 and S2

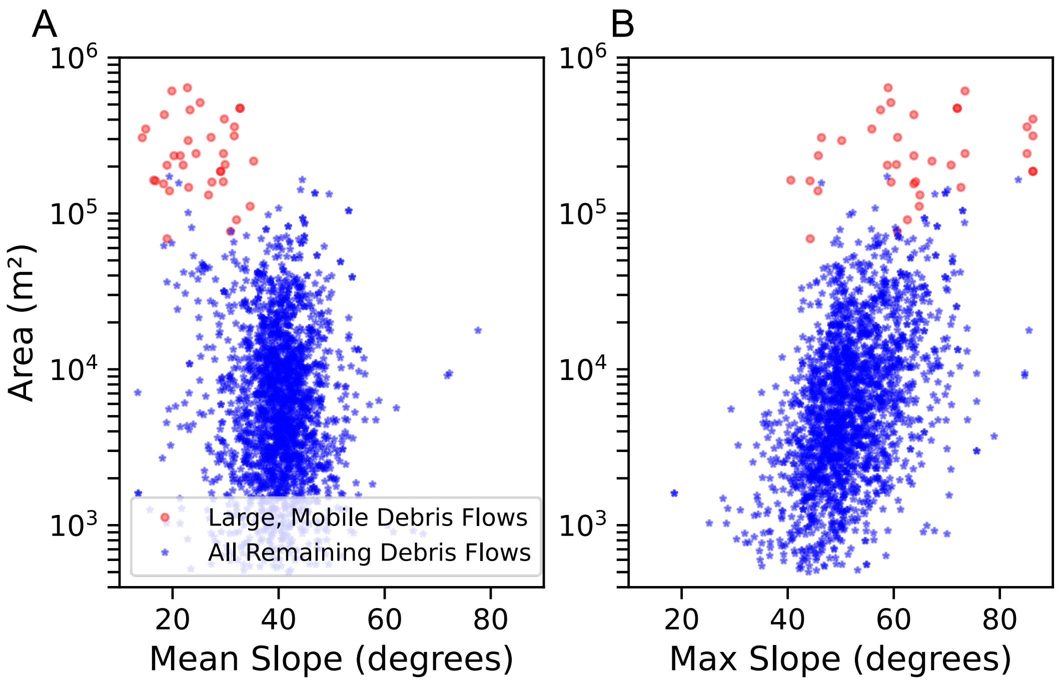

Fig. S1.

Mean and maximum slope against debris flow area for large, mobile debris flows and all remaining debris flows within the empirical inventory from Wenchuan. Slope information is obtained from the 30 m JAXA DEM.

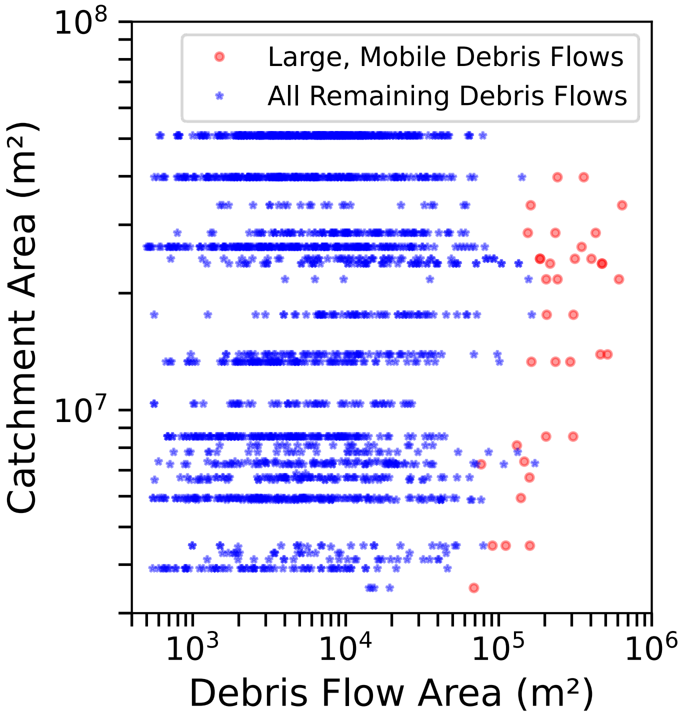

Fig. S2.

Catchment area against debris flow area for large, mobile debris flows and all remaining debris flows within the empirical inventory from Wenchuan.

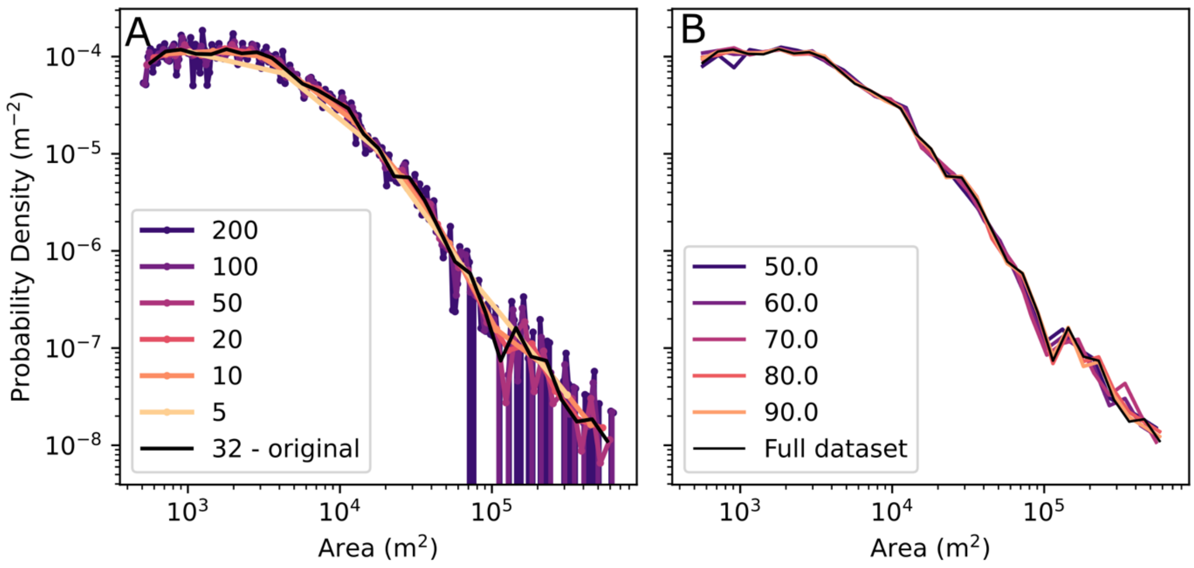

Fig. S3.
Probability density functions for debris flow area using the Wenchuan debris flow inventory. In Panel A, we vary the number of logarithmic bins used to segment the dataset from 5 to 200. The black line shows the data segmented into 32 bins as advised by the *powerlaw* Python package. The change in frequency at ~10^5 m^3 is evident in most instances, excluding when using only 5 bins. In Panel B, we vary the proportion of the data used to derive the probability density curve from 50% to 100% (black line).

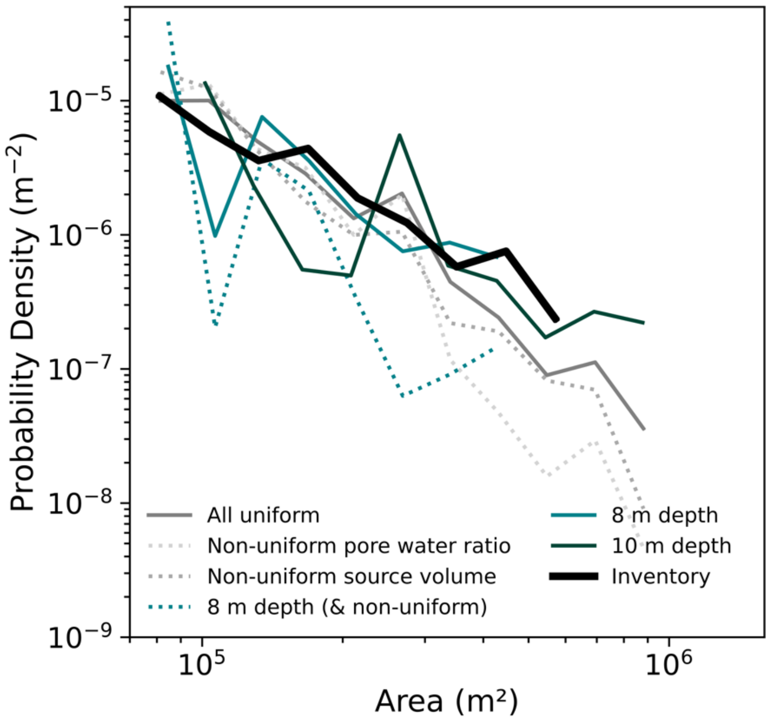

Fig. S4.

Magnitude-frequency distributions for a selection of simulated debris flow inventories generated using the Monte Carlo Simulation and Massflow model runs. Dotted lines represent when a non-uniform distribution has been used to infer either the source volume or the pore water ratio (degree of bed saturation). The Wenchuan inventory dataset is shown by the solid black line.

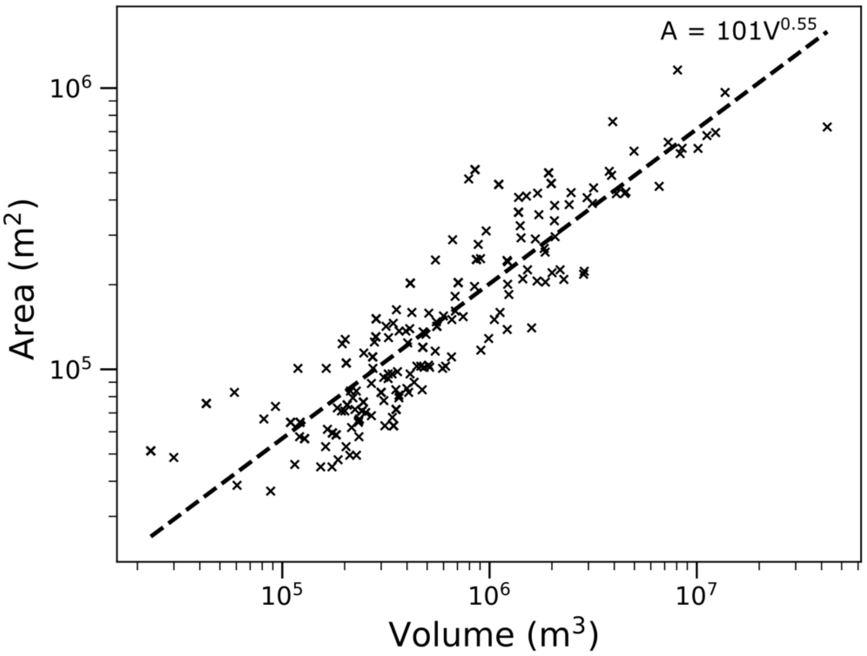

Fig. S5.
Area-volume scaling relationship for debris flows simulated in Massflow. The best fitting line has an r^2 value of 0.66.

Table S1. Set-up for each Monte Carlo Simulation. The combination of distributions used to represent each input parameter for each Monte Carlo Simulation. Ten Monte Carlo Simulations were run in total.

	**Pore water ratio (0 to 1.0, 0.1 increments)**	**Source Volume (24 300 m^3 , 97 200 m^3, 172 800 m^3 and 270 000 m^3)**	**Maximum Entrainable Sediment Depth (4 m to 10 m, 1 m increments)**
**Run 1**	Uniform	Uniform	Uniform
**Run 2**	Lognormal	Uniform	Uniform
**Run 3**	Uniform	Inverse Gamma	Uniform
**Run 4**	Lognormal	Inverse Gamma	Uniform
**Run 5**	Lognormal	Inverse Gamma	10 m
**Run 6**	Uniform	Uniform	10 m
**Run 7**	Uniform	Uniform	6 m
**Run 8**	Uniform	Uniform	8 m
**Run 9**	Lognormal	Inverse Gamma	8 m
**Run 10**	Lognormal	Inverse Gamma	6 m

Table S2. The input parameters for Massflow used in this study to model debris flows in the Luoquanwan catchment.

ρ_1	ρ_w	c	C_z	λ	$\varphi_{voellmy}$	δ	ϕ_2
2020 kg m^{-3}	1000 kg m^{-3}	2900 Pa	12	0 to 1.0	12°	20°	35°
